# Maillard and Hydrolytic Reactions in Subcritical Water Extraction of Bioactive Compounds from Licorice

**DOI:** 10.3390/molecules27206851

**Published:** 2022-10-13

**Authors:** Rui Fan, Yanxiang Gao

**Affiliations:** 1 Department of Nutrition and Food Hygiene, School of Public Health, Peking University, Beijing 100191, China; 2 Beijing Key Laboratory of Functional Food from Plant Resources, College of Food Science & Nutritional Engineering, China Agricultural University, Beijing 100083, China

**Keywords:** glycyrrhizic acid, glycyrrhetinic acid, glycyrrhetinic acid 3-*O*-mono-β-D-glucuronide, antioxidant, Maillard reaction

## Abstract

Nowadays, subcritical water extraction (SWE) techniques are extensively investigated worldwide, while the thermal reactions that inevitably occur under subcritical water conditions are rarely studied. In order to investigate the behaviors of the different reactions during SWE of bioactive compounds from licorice, the Maillard reaction process was accessed via their products and the hydrolytic reaction was analyzed according to the kinetic parameters. In addition, the contents of total phenolics and flavonoids in the extracts obtained at the different temperatures were determined and total antioxidant capacities were evaluated by HPLC-ABTS^+^. The results showed that flavonoids and phenolics from licorice as well as new compounds generated via the Maillard reaction contributed to the antioxidant activity of the extracts. The fluorescence, color and absorbance of the extracts showed that the degree of the Maillard reaction increased with the rise of the extraction temperature. The kinetics of extraction for glycyrrhizic acid showed that it was firstly extracted by diffusion, and then was hydrolyzed into glycyrrhetinic acid 3-*O*-mono-β-D-glucuronide and glycyrrhetinic acid following a first-order mechanism. These findings could provide deep insights into the SWE process and a new method for producing glycyrrhetinic acid 3-*O*-mono-β-D-glucuronide and glycyrrhetinic acid.

## 1. Introduction

Licorice, the roots of *Glycyrrhizia glaubra*, has been widely used in medicine for treating phthisis (pulmonary tuberculosis), contagious hepatitis, and bronchitis because of its anti-inflammatory, anti-viral, anti-allergic, anti-oxidant, gastro-protective, and anti-cancerous properties [1]. Triterpene saponins and flavonoids have been demonstrated to be major constituents and show several pharmacological activities. Triterpenoid saponins are also sweet-tasting [2].

Water reaches a subcritical state as the temperature rises; during this period, water properties exhibit greater changes, including decreases in viscosity, surface tension, and dielectric constant and an increase in diffusivity, which results in a significant increase in the solubility of components with less polarity. Therefore, subcritical water extraction (SWE) technology possesses a series of advantages: more efficiency and selectivity, lower costs, and less pollution [3]. In fact, the full utilization of subcritical water technology is not only used for extraction but also for the chemical reaction that occurs due to the increasing self-ionization of water at higher temperatures [4,5]. It was expected that SWE technology can be used to extract bioactive compounds from licorice, i.e., single bioactive substances including glycyrrhizic acid and liquiritin [6,7]. The chemical reaction that takes place during SWE from licorice has been neglected. Examples of glycyrrhizic acid originally present in the matrices might be released and interacted, and new compounds might be generated [8,9]. Based on our previous research, it was speculated that the Maillard reaction might occur in subcritical water due to a reduced pH value as well as the formation of 5-hydroxymethyl furfural [10]. Therefore, only with a sufficient understanding of the SWE process can we utilize this technology with high efficiency and prepare high-value-added products directionally.

**Figure 1:**
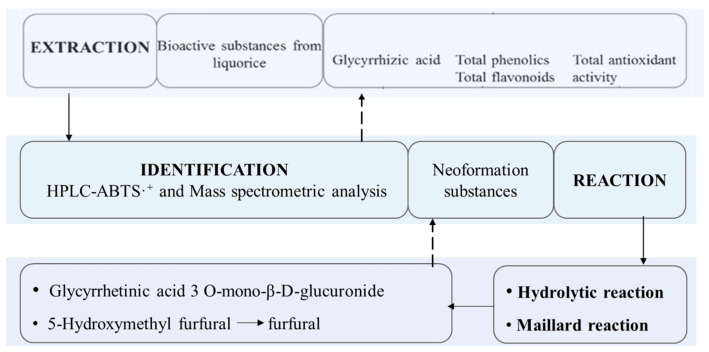
The study design of the thermal reactions in subcritical water extraction of bioactive compounds from licorice.

In order to compensate for the lack of research on what happens during the SWE process of multiple bioactive substances from licorice, we focused on the reaction process, including the Maillard reaction and thermal hydrolysis. We originally investigated the effect of the extraction temperature and time on the extraction behaviors of bioactive substances and its antioxidant activity by HPLC-ABTS^+^, and then analyzed the Maillard and hydrolysis reaction process under SW, especially hydrolysis reaction kinetic of glycyrrhizic acid, which has been firstly discovered that glycyrrhizic acid can transform into glycyrrhetinic acid 3-O-mono-β-D-glucuronide under SW (study design in Figure 1). This work clarifies the SWE process and provides a clue to preparing value-added substances extracted from licorice.

## 2. Results and Discussion

### 2.1. Subcritical Water Extraction

#### 2.1.1. Effect of Extraction Temperature on the Content of Bioactive Compounds in the Extract

**Figure 2:**
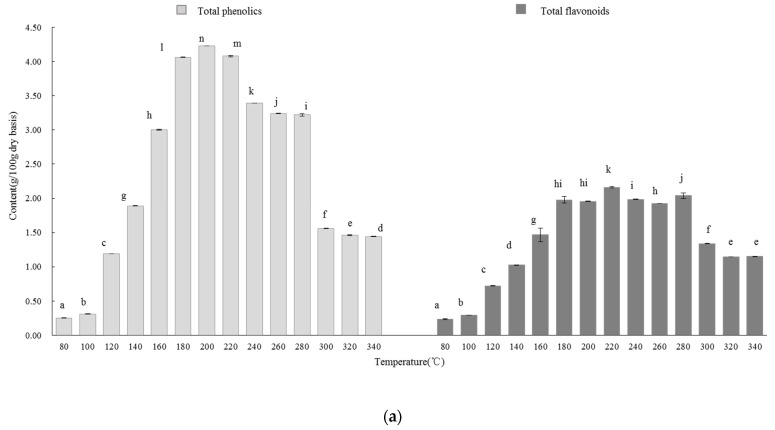

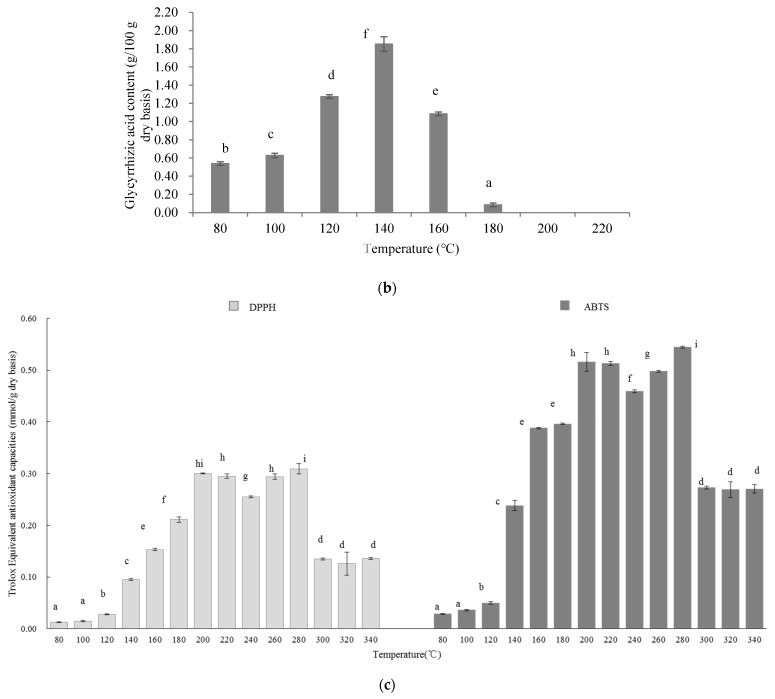
The effect of extraction temperature on the content of total flavonoids, total phenolics (**a**) and glycyrrhizic acid (**b**) and the effect of extraction temperature on total antioxidant activity (**c**). The results are expressed as the mean ± standard deviation (*n* = 3); different letters indicate significant differences (*p* < 0.05) and the same letters indicate insignificant differences.

The SWE of bioactive compounds from licorice was carried out at different temperatures (80–340 °C) for 20 min with a solid-to-water ratio of 1:30 at pH 8 under 7 MPa. As described in Figure 2a, the effects of extraction temperature on the content of total phenolics and total flavonoids were significant (*p* < 0.05). Total phenolic content increased with a rise in temperature from 80 to 200 °C and then decreased. The maximum content of total phenolics was 4.213 g/100 g dry basis at 200 °C, which was 15-fold higher than that obtained at 80 °C. The content of total flavonoids increased significantly (*p* < 0.05) from 0.297 g/100 g dry basis at 80 °C to 2.021 g/100 g dry basis at 220 °C. With the rise in temperature, the water dielectric constant (which is normally 80) considerably decreases (to approximately 36.5 at 190 °C, 33 at 210 °C), presenting a behavior similar to organic solvents (such as methanol, whose dielectric constant is 32.6 at 25 °C) and allowing a faster extraction and a better extraction yield for phenolic compounds and flavonoids [11]. In addition, the major barrier to releasing polyphenol compounds from plant materials was the cell wall, rich in cellulose and pectin, and SW was used to facilitate cell-wall destruction [12]. The existing bonds (ester and/or ether bonds) between lignin and cellulose could be effectively hydrolyzed in SW; consequently, phenolic compounds were released [13]. Therefore, SW could enhance the extraction recovery of flavonoids and phenolics. However, the content of total phenolics and flavonoids started to decrease from 200 and 220 °C, respectively. It was speculated that these compounds further degraded and reacted with other components [11]. It was clearly observed that the maximum extraction yield of total flavonoids and phenolics depending on the temperatures were different, i.e., 220 vs. 200 °C. A possible reason may be that multiple single substances belong to total flavonoids and total phenolics with unique structures. Some kinds of phenolic compounds contain abundant phenolic hydroxyl groups, which lead to relatively more polarity than flavonoid compounds, which exhibit many (large) carbon atoms (≥15) and nonpolar functional groups such as -OCH3 (e.g., isorhamnetin) and -H (e.g., kaempferol) [14]. Therefore, it is possible that flavonoids show less polarity than some phenolics, which generates the maximum extraction yield of total flavonoids at higher temperatures. A similar phenomenon was reported by Kumaret al. (2011) [15]. In addition, our previous research reported that flavonoids in licorice contained flavanones (liquiritin apioside, liquiritin), chalcones (isoliquiritin apioside, licuraside), flavonols (isolicoflavonol), isoflavone (ononin), and flavone glycosides (licorice-glycoside A, licorice-glycoside B) [10]. The structures are listed in the Supplementary Materials of that study. It was observed that some flavonoids contain nonpolar side chains, such as alkyl groups (isolicoflavonol) and aglycones (licorice-glycoside A, licorice-glycoside B). According to other reports [16,17,18], there are some prenylated flavonoids with less polarity, including lupalbigenin, glabrene, gancaonin F, and gancaonin G, in licorice extracts, which are more stable than phenolics. Therefore, compared with the optimal extraction temperature for total phenolics, the optimal extraction temperature for total flavonoids was relatively higher.

As shown in Figure 2b, the glycyrrhizic acid content in the extract was affected significantly (*p* < 0.05) by the extraction temperature. With the rise in extraction temperature, glycyrrhizic acid content increased significantly (*p* < 0.05). The maximum content was 1.834 g/100 g dry basis at 140 °C. Further increasing the temperature led to a sharp decrease in the content, and no glycyrrhizic acid was detected over 200 °C, which was attributed to its instability and decomposition [19,20]. This decomposition could be reflected by glycyrrhetinic acid 3-O-mono-β-D-glucuronide and glycyrrhetinic acid formation (Figure 2b and Figure 5b,c). Glycyrrhetinic acid 3-*O*-mono-β-D-glucuronide, which does not exist naturally in licorice, is formed after cleaving the distal glycosidic bond of glycyrrhizic acid. In fact, the decomposition reaction of glycyrrhizic acid occurred at a certain temperature, coupled with some exposure time for sufficient energy. We observed that the decomposition of glycyrrhizic acid occurred above 100 °C, i.e., 100 °C for 70 min vs. 120 °C for 50 min, as indicated in our previous report in *Food Chemistry* [21]. In this study, the decrease in glycyrrhizic acid content was observed to begin from 140°C, as shown in Figure 2b. However, this does not mean that the decomposition occurred from 140 °C. As long as the accumulated energy is sufficient, extraction and decomposition may take place simultaneously. A possible reason for the decrease in glycyrrhizic acid above 140 °C in this study was the higher temperature, which provided sufficient energy for glycyrrhizic acid hydrolysis to occur rapidly, which then lead to the decomposition rate increasing beyond its accumulation rate.

Based on the previous report [10], the contents of total flavonoids and phenolics compounds were 2.037 and1.932 g/100 g dry basis by the traditional extraction with 50% ethanol at 80 °C for 5 h, which was equal with the contents in this research with subcritical water at 140 °C for 20 min. Under the same conditions, the content of glycyrrhizic acid in the extract was 1.834 g/100 g dry basis, which was 1.5 times that of the extract obtained with water at 80 °C for 5 h. Therefore, the extraction of bioactive substances with subcritical water showed the high efficiency and low energy consumption.

#### 2.1.2. Effect of Extraction Temperature on the Total Antioxidant Activity of Licorice Extract

The total antioxidant activity of licorice extract was evaluated with DPPH and ABTS assays. As shown in Figure 2c, total antioxidant activity was significantly affected (*p* < 0.05) by extraction temperature, and the total antioxidant activity, evaluated by two assays, increased significantly (*p* < 0.05) with the rise in extraction temperature from 80 to 280 °C. The higher antioxidant activities of the extract were obtained between 200 and 280 °C. At 280 °C, the highest values were 0.544 mmol TEAC/g dry basis with an ABTS assay and 0.309 mmol TEAC/g dry basis with a DPPH assay, respectively.

Compared with the curve in Figure 2a,c, there were some differences between the content of total phenolics, total flavonoids, and total antioxidant activity from 240 to 280 °C. In order to estimate the relationship between the total antioxidant activity and the total phenolic and flavonoid content, correlation analysis was carried out (Table S1 Supplementary Materials). The linear correlation coefficients (*R*^2^ > 0.90) between 80 and 240 °C showed that there was a good relationship between the content of total flavonoids, phenolics, and the antioxidant capacity, which indicated that the antioxidant activity could be attributed to the phenolics and flavonoids in the extract. This result is in significant agreement with a previous report [22]. However, at higher temperature (>240 °C), phenolics and flavonoids were decomposed, and the extract still exhibited strong antioxidant activity. The *R*^2^ value between 240 and 280 °C was relatively low (*R*^2^ < 0.85), and there was even a negative correlation between total flavonoids, total phenolics and antioxidant capacity, which indicated that some non-phenolic compounds were generated at a higher temperature (>240 °C). They provided a more effective contribution to the antioxidant activity and were the main antioxidants [23].

### 2.2. HPLC-ABTS^+^ Antioxidant Activity Evaluation and HPLC-MS/MS and UPLC-MS Identification of Bioactive Compounds in Licorice Extract

**Figure 3:**
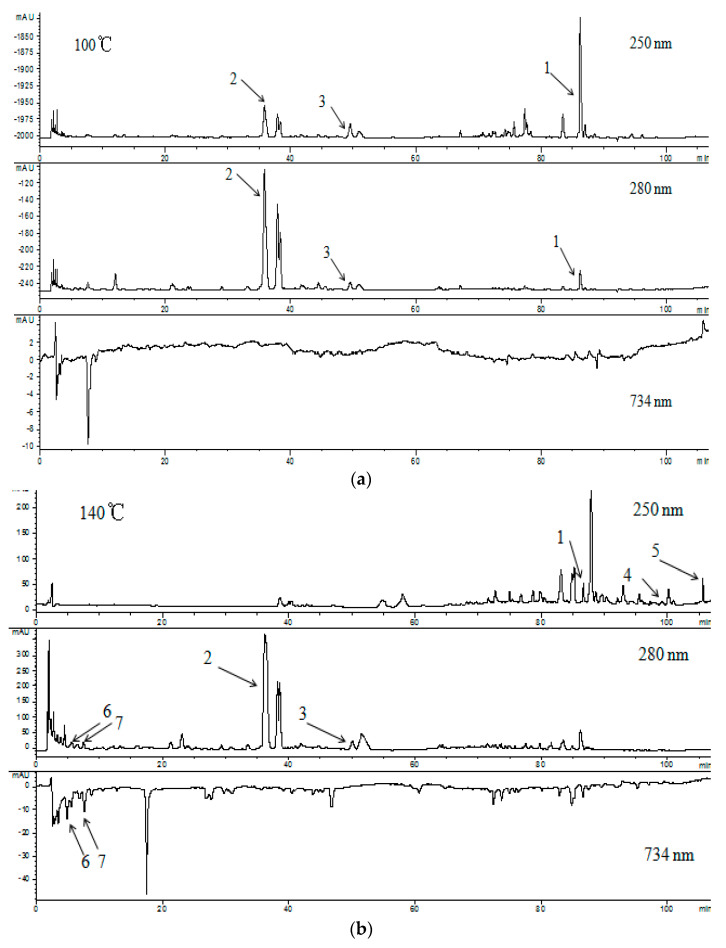

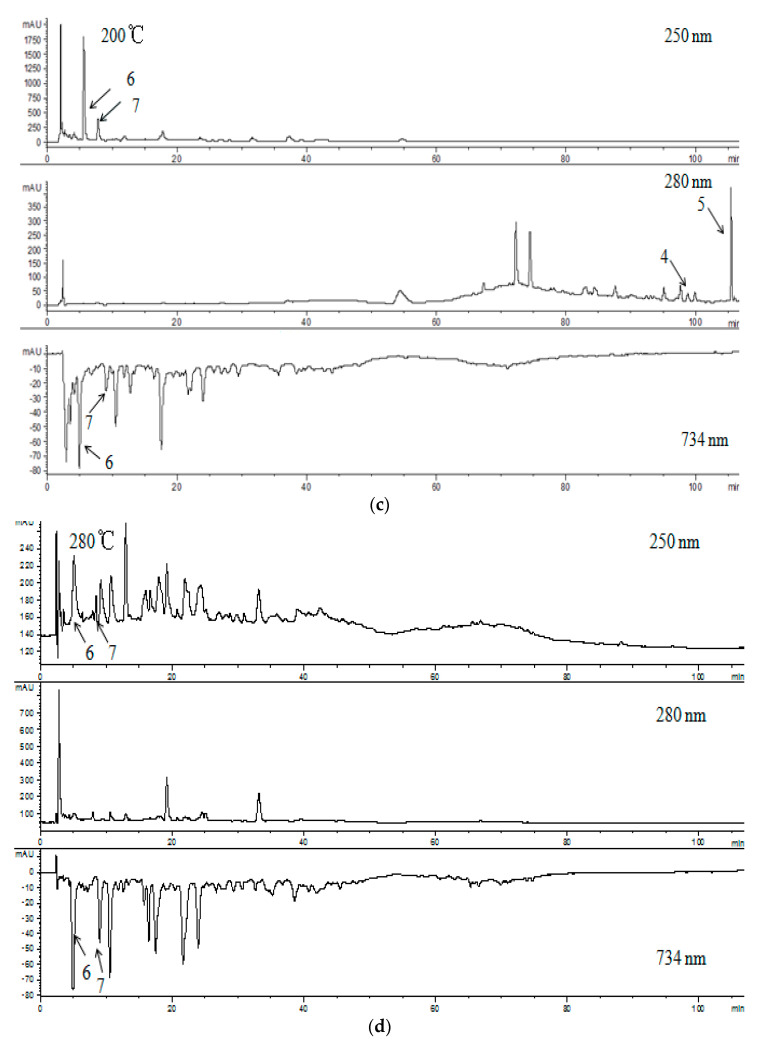

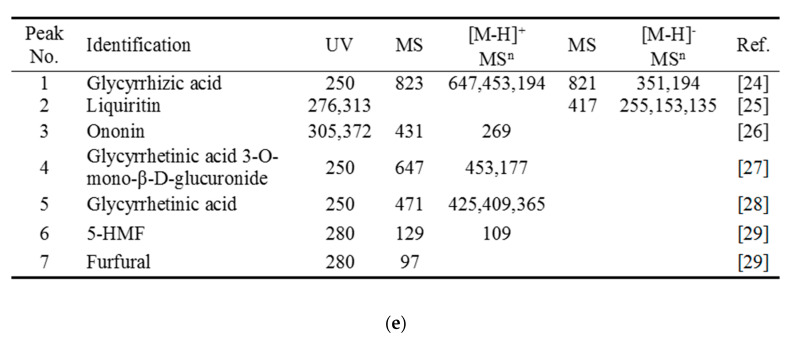
HPLC-ABTS·^+^ chromatographic profiles and HPLC-MS/MS information regarding the extracts at different temperatures. (**a**–**d**) was the chromatographic profiles at 100, 140, 200 and 280 °C; (**e**) the HPLC-MS/MS information [24,25,26,27,28,29].

The antioxidant activities of the licorice extracts were evaluated with the HPLC-ABTS·^+^ technique, and the results are shown in Figure 3. At 100 °C, Compounds 1, 2, and 3 were clearly found. With the rise in extraction temperature, some compounds were newly generated, such as Compounds 6 and 7, and their chromatographic profile peak areas at 250 nm and 280 nm increased from 140 to 200 °C. Accordingly, their corresponding chromatographic profile peak areas at 734 nm also increased, indicating that Compounds 6 and 7 exhibited strong antioxidant activities. In addition, Compounds 4 and 5 were newly formed at 140 °C, and the chromatographic profile peak areas of Compounds 4 and 5 increased at 200 °C; however, Compound 1 could not be observed. At a higher temperature (280 °C), Compounds 1, 2, 3, 4, and 5 disappeared. This phenomenon could indicate that there were some reactions during the process of SWE.

The bioactive compounds in the licorice extract were identified with HPLC-MS/MS. The information of MS and UV is shown in Figure 3. These identified compounds were confirmed by comparing them with the standards using UPLC-MS/MS (Figure S1). In terms of these identified compounds, it is proposed that the Maillard reaction and hydrolysis of glycyrrhizic acid occurred in SW.

### 2.3. Analysis of Non-Specific Indicators of Maillard Reaction

#### 2.3.1. Total Reducing Sugars and Total Free Amino Acid Content

**Figure 4:**
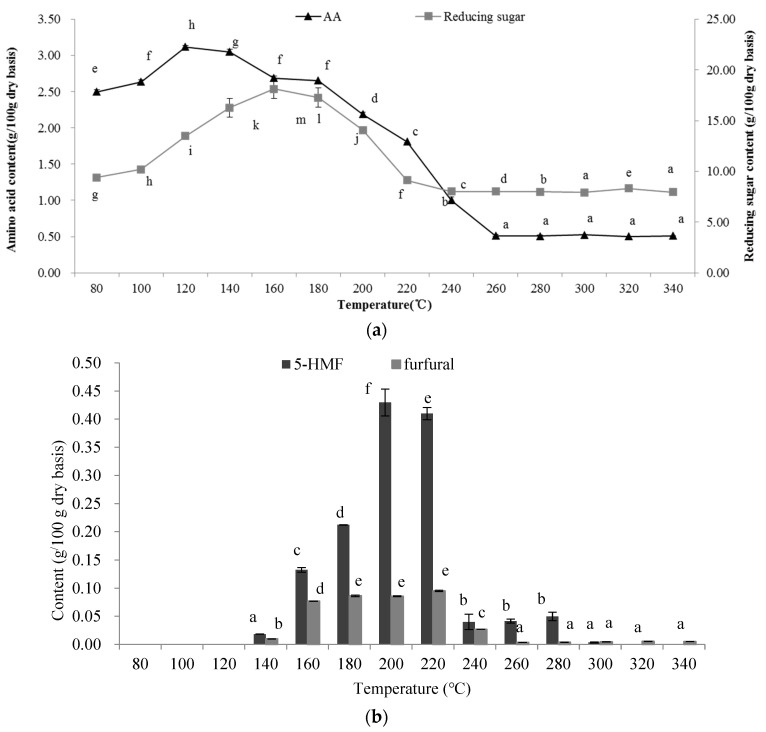
The analysis of indicators of Maillard reaction in the extracts at different temperatures, (**a**) total reducing sugars and total amino acids contents; (**b**) 5-HMF and furfural contents. The results are expressed as the mean ± standard deviation (*n* = 3); different letters indicate significant differences (*p* < 0.05), and the same letters indicate insignificant differences.

The Maillard reaction is initiated by the reducing-end carbonyl group and the free primary amine group, and most of these groups are derived from reducing sugars and free amino acids in foodstuffs, respectively. As shown in Figure 4a, the total reducing sugars and free amino acid concentrations were significantly (*p* < 0.05) affected by extraction temperature. Free amino acid content increased and reached a maximum level at 120 °C and then decreased sharply. The total reducing sugar content increased from 80 to 160 °C and then decreased at the higher temperature. The effects of the extraction temperature on the content of reducing sugars and free amino acid were determined by their solubility and consumption. On the one hand, licorice contains a large number of polysaccharides (such as cellulose and hemicellulose) and crude protein [19]. With the rise in temperature, the ionic product constant (*K*w) of SW increases, which leads to a high concentration of H^+^ and OH^−^ catalyzing polysaccharide and protein that hydrolyzes into small molecular compounds such as monosaccharides and amino acids, respectively, so reducing sugars and amino acids were released [30]. Accordingly, with the rise in temperature, reducing sugars and free amino acids were inevitably involved in the Maillard reaction during SWE, leading to the reduction in their content. In addition, it was also observed that there was a certain degree of correlation between the fluorescence data and the amino acid content (*R*^2^= 0.788 for λ_Em_ 460, *R*^2^= 0.752 for λ_Em_ 528). Therefore, the Maillard reaction obviously occurred.

#### 2.3.2. Color Measurement

The color of the extract is the result of colored natural products associated with the raw material and the color compounds generated during processing [31]. Therefore, it is useful to study browning in relation to variations in the color of the licorice extract. The results are shown in Table 1. For the value of L, the value decreased with the elevating temperature, and the L value was significantly lower at temperatures ranging from 80 °C to 120 °C than those above 120 °C (*p* < 0.05). As can be seen, the a* value increased with the elevating temperature. Compared with the relatively lower temperature, i.e., 80–120 °C, the a* value increased significantly from 180 °C (*p* < 0.05), while the changes in the a* value were insignificant at different temperatures from 180 °C to 340 °C (*p* > 0.05). The value of a* tended to increase with more severe treatment, where the a* value clearly shifted towards the red component, corresponding to Maillard reaction development [32]. As expected, the a* value in the red component increased with an elevating extraction temperature, which indicated that the higher the extraction temperature was, the greater the degree of Maillard reaction that developed. In addition, a negative correlation was presented between L* and a* (*R*^2^ = 0.878). The b* value always increased with elevating temperature. Compared with the b* value at 80 °C, the b* value significantly increased from 260 °C (*p* < 0.05), and the b* value at 280 °C was significantly larger than those at relatively lower temperatures from 80 °C to 140 °C (*p* < 0.05). The positive correlation between a* and b* (*R*^2^ = 0.746) was also testified. Therefore, the color alteration of the extract indicated the occurrence of the Maillard reaction during SWE. The YI value, the integrated index for the browning degree, kept increasing as the temperature increased, with a significant increase observed from 120 °C (*p* < 0.05), but this increase was insignificant above 180 °C (*p* > 0.05), which indicated a higher degree of Maillard reaction.

#### 2.3.3. Absorbance Measurement

As described previously, the absorbance measurement was performed at 280, 360, and 420 nm, corresponding to early, advanced, and final Maillard reaction product appearance in the extract. As shown in Table 1, a significant (*p* < 0.05) increase in absorbance at 280 nm for the extract was observed from 120 to 180 °C, and the decrease occurred at a higher temperature (>180 °C). The value of A_260_ nm represents the primary products during the Maillard reaction, which revealed that the Maillard reaction likely occurred from 120 °C, and the decreasing values from 280 °C might be due to the primary products reacting with other substances, which we speculated was the point at which the Maillard reaction moved into the next stage. The absorbance at 360 nm for the extract increased from 120 to 220 °C (*p* < 0.05) and then sharply decreased (*p* < 0.05), which implied that advanced products were generated in the process and that degradation occurred at 220 °C. This phenomenon was in agreement with a report by Kim [33]. Brown color development (*A*_420_) is the easiest measurable consequence of a Maillard reaction, as it offers a visual estimation. As shown in Table 1, the browning intensity of the extracts increased significantly with the temperature which increased from 80 °C to 260 °C (*p* < 0.05). Compared with the *A*_420_ value at temperatures below 120 °C, it was obviously larger at other temperatures (*p* < 0.05), which was consistent with our visual observations.

### 2.4. Analysis of Specific Indicators of Maillard Reaction

#### 2.4.1. Determination of Fluorescent Advanced Glycated End Products

As a result of the Maillard reaction, newly formed compounds exhibit a strong emission between the wavelengths of 400 and 600 nm when excited at a wavelength of 360 nm [23]. As shown in Table 1, the change trends between the two emission wavelengths are the same. Compared with the fluorescence values of the extracts obtained at temperatures below 140 °C, the fluorescence values increased significantly (*p* < 0.05) over 120 °C, indicating that the Maillard reaction might have occurred from 120 °C, and the level of fluorescence reached the maximum at 280 °C, indicating that the formation of fluorescent Maillard reaction products was favored at 280 °C, and the fluorescence values then decreased significantly (*p* < 0.05), due to the polymerization of advanced glycated end products and some reactions with other components [23].

#### 2.4.2. Determination of 5-Hydroxymethyl Furfural and Furfural

The compound 5-Hydroxymethyl furfural(5-HMF) is an intermediate compound in the Maillard reaction process. As shown in Figure 4b, 5-HMF was not detected in the extract obtained at temperatures lower than 120 °C. However, elevating the extraction temperature gave rise to the formation of 5-HMF, which reached 0.429 g/100 g dry basis at 200 °C. These results are in agreement with previous findings, which indicate the occurrence of a Maillard reaction during SWE at higher extraction temperatures [34]. However, the 5-HMF content decreased over 200 °C, because 5-HMF, an intermediate product in the Maillard reaction, can also participate in the degradation, polymerization, and reactions with other components [11,32].

Furfural is another compound formed by the degradation of ascorbic acid, sugars, and Amadori compounds [35]. With the rise in temperature, the furfural content in the extract increased significantly (*p* < 0.05). A higher content was obtained between 160 and 220 °C. However, the furfural content decreased at temperatures higher than 220 °C because the furfural had a high activity and participated in further reactions. In the advanced stage of the Maillard reaction, a series of reactions took place, including cyclizations, dehydrations, retroaldolizations, rearrangements, isomerizations, and further condensations, which ultimately led to the formation of brown nitrogenous polymers and copolymers, known as melanoidins [36].

### 2.5. Thermal Hydrolysis of Glycyrrhizic Acid

**Figure 5:**
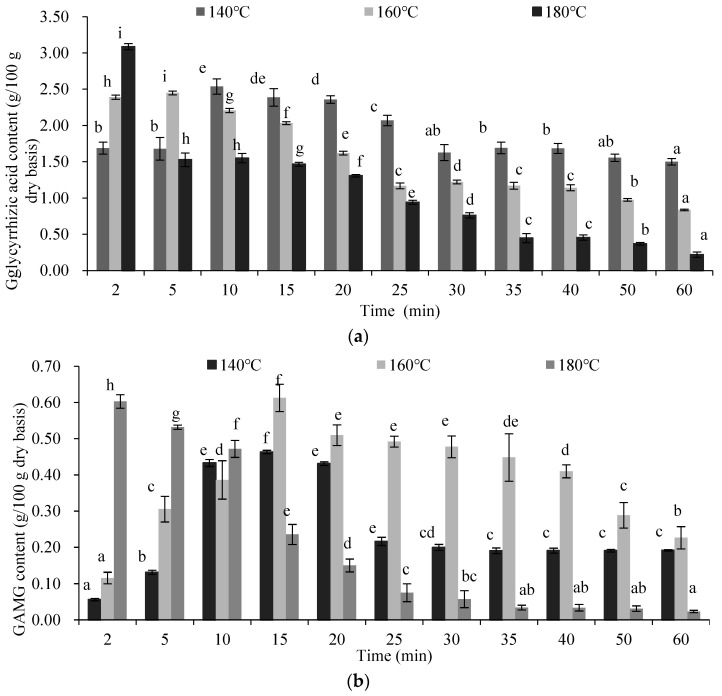

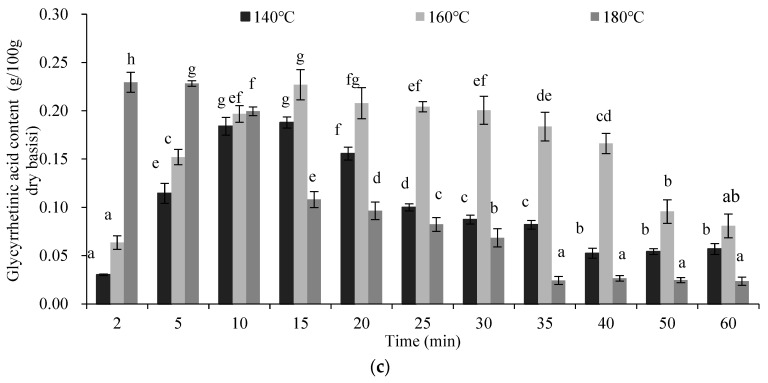
Thermal hydrolysis of glycyrrhizic acid under different temperatures and times, (**a**) the content of glycyrrhizic acid; (**b**) the content of GAMG; (**c**) the content of glycyrrhetinic acid. GAMG: glycyrrhetinic acid 3-*O*-mono-β-D-glucuronide.The results are expressed as the mean ± standard deviation (*n* = 3); different letters indicate significant differences (*p* < 0.05) and the same letters indicate insignificant differences.

The thermal hydrolysis of glycyrrhizic acid at different temperatures was demonstrated, and the results are shown in Figure 5a. Glycyrrhizic acid was hydrolyzed and formed glycyrrhetinic acid and glycyrrhetinic acid 3-*O*-mono-β-D-glucuronide in SW, and this study is the first to find these. Glycyrrhizic acid content increased before 10 min at 140 °C and before 5 min at 160 °C, which implies that the decomposition of glycyrrhizic acid hardly occurred. This result might be due to an amount of energy that is insufficient for breaking the glucosidic bond. SWE only promoted the accumulation of glycyrrhizic acid at a lower temperature over a short period. With the extension of the extraction time, glycyrrhizic acid content decreased significantly (*p* < 0.05), which implied the decomposition of glycyrrhizic acid. However, at a higher temperature (180 °C), glycyrrhizic acid decreased sharply (*p* < 0.05) at the beginning of the extraction, which indicated that glycyrrhizic acid was decomposed.

As shown in Figure 5b,c, the content of glycyrrhetinic acid 3-*O*-mono-β-D-glucuronide and glycyrrhetinic acid in the extract increased with the extension of extraction time at 140 and 160 °C. Glycyrrhetinic acid 3-*O*-mono-β-D-glucuronide content increased significantly (*p* < 0.05) after 5 min of extraction at 140 °C, reached a maximum value at 15 min, and then decreased due to its decomposition, hydrolyzing one molecule of glucuronic acid to form glycyrrhetinic acid. At 180 °C, the glycyrrhetinic acid 3-*O*-mono-β-D-glucuronide content of the extract obtained in 2 min decreased, due to its transformation and/or decomposition.

As shown in Figure 5c, glycyrrhetinic acid content increased over a short period at lower temperatures (140 °C and 160 °C). It was efficiently extracted from licorice with SW. The higher content of the extract obtained at 180 °C was due to the conversion from glycyrrhizic acid and glycyrrhetinic acid 3-*O*-mono-β-D-glucuronide. Therefore, the glycyrrhetinic acid yield in the extract was due to the GA in the licorice coupled with the transformation from glycyrrhizic acid and glycyrrhetinic acid 3-*O*-mono-β-D-glucuronide in the SW.

### 2.6. Kinetic Analysis

**Figure 6:**
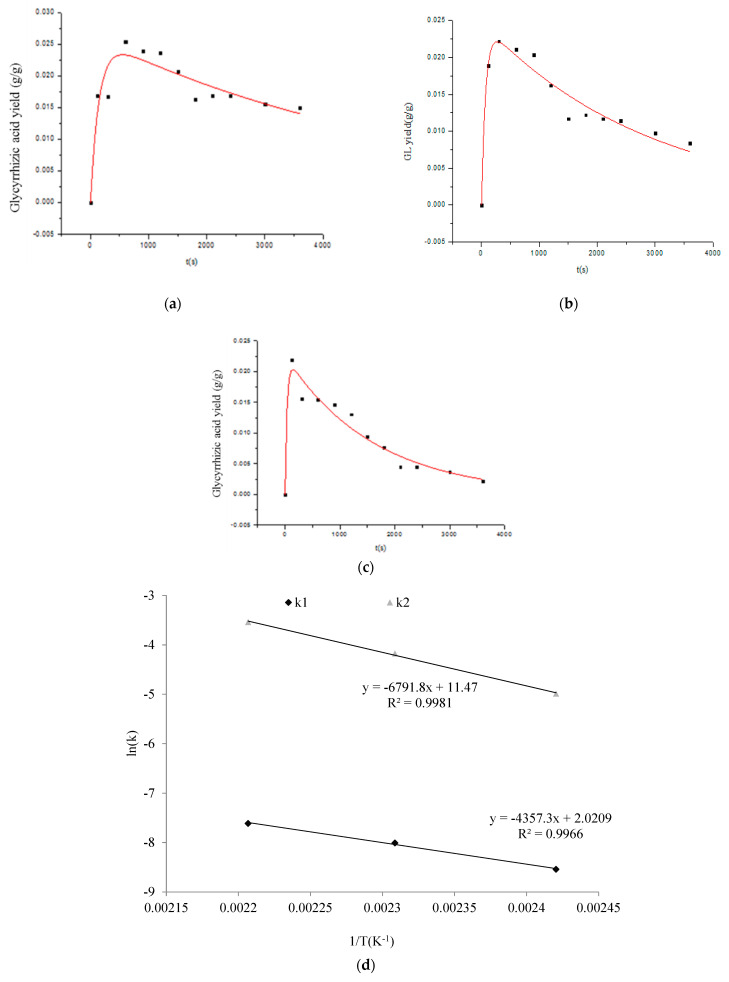
The kinetic parameters at different temperatures, (**a**–**c**) the fitting curve of the extraction and hydrolytic rate constant at 140 °C, 160 °C, and 180 °C, respectively; (**d**) the Arrhenius plot.

The kinetic analysis of glycyrrhizic acid extraction and hydrolysis in SW was based on consecutive steps. Glycyrrhizic acid was first extracted from inside of the licorice powder by diffusion following a first-order mechanism. Glycyrrhizic acid in the extract was then subjected to a hydrolytic reaction following the first-order mechanism due to the extension of the extraction time under a high temperature and pressure. Figure 6a–c shows the results of the time course of the glycyrrhizic acid yield from 2 to 60 min at different temperatures (140–180 °C). The experimental data were well (*R*^2^ > 0.85) correlated with the irreversible, consecutive, unimolecular-type first-order mechanism using Equation (9). The results showed that the extraction and hydrolytic rates increased with the rise in extraction temperature, which is in agreement with previous research [21]. As the temperature increased from 140 to 160 °C, *k_2_* increased significantly (*p* < 0.05) from 0.00683 s^−1^ to 0.0141 s^−1^, which indicated that the energy barrier for cleaving the glycosidic bond was sufficient, which led to the fast hydrolytic reaction rate [21].

Figure 6d shows the Arrhenius plots for the SWE process. The Arrhenius plots with the pre-exponential factor and activation energy are shown in Figure 6d. The Arrhenius equations are expressed as follows:

$$\ln k_{1}=-\frac{36.17}{RT}+\ln7.55$$

$$\ln k_{2}=-\frac{54.46}{RT}+\ln9.6\times 10^{4}$$

Based on these values, an activation energy below 40 kJ/mol generally indicated a diffusion-controlled process, and higher values represented a chemical reaction process [37]. In terms of *E*a, the diffusion-controlled process was governed by the mass transfer or diffusion of the aqueous phase from the solid reactant to the bulk solution. Accordingly, the obtained activation energy (36.17 kJ/mol) clearly indicated a diffusion control mechanism for the glycyrrhizic acid extraction process. The *E*a value of the extraction was smaller compared to that of the hydrolysis, which indicated that the hydrolysis of glycyrrhizic acid requires more energy to cleave the glycosidic bond. These results could explain why a high extraction temperature was conducive to a hydrolytic reaction that overcomes the energy barrier.

## 3. Materials and Methods

### 3.1. Chemicals and Plant Material

#### 3.1.1. Chemicals

The 1,1-Diphenyl-2-picrylhydrazyl (DPPH), 2,2’-Azinobis (3-ethylbenzothiazoline-6-sulfonic acid) diammonium salt (ABTS) and Folin–Ciocalteu reagent were obtained from Sigma (St. Louis, MI, USA). Standards of glycyrrhizic acid, ononin, glycyrrhetinic acid, and liquiritin were purchased from Winherb Medical Co., Ltd. (Shanghai, China). The 5-HMF and furfural were purchased from Aladdin Industrial Co., Ltd. (Shanghai, China). Glycyrrhetinic acid 3-*O*-mono-β-D-glucuronide (≥85%) was provided from the laboratory of biotechnology in College of Life Science of Beijing Institute of Technology. All the reagents (HPLC grade) were obtained from Merck (Shanghai, China).

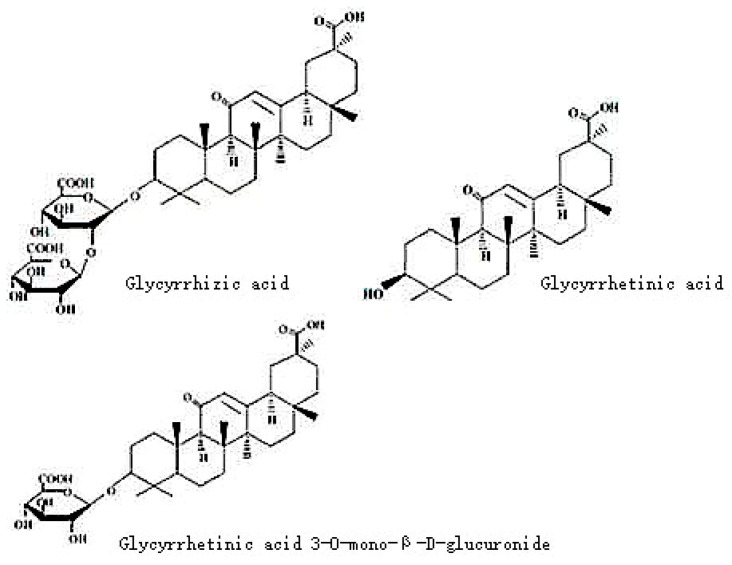



#### 3.1.2. Plant Material

Licorice roots were obtained from Pufansheng Biotechnology Company (Inner Mongolia, Huhehaote, China) and ground to an average particle size below 0.4 mm by a high-speed mill (Model HY-200; Beijing Huanya Scientific Ltd., Beijing, China). Moisture content was determined by drying samples at 100 ± 0.5 °C to a constant weight, and the moisture measured was 4.05%. The lipid content was 5.23 ± 0.36% as determined by Soxhlet apparatus with hexane for 4 h. In addition, the essential oil in licorice was extracted with the hydro-distillation method based on the recommendation from the *Pharmacopoeia of the People’s Republic of China*, and the essential oil content was 0.04 ± 0.06%.

### 3.2. Apparatus and Extraction Procedure

**Figure 7:**
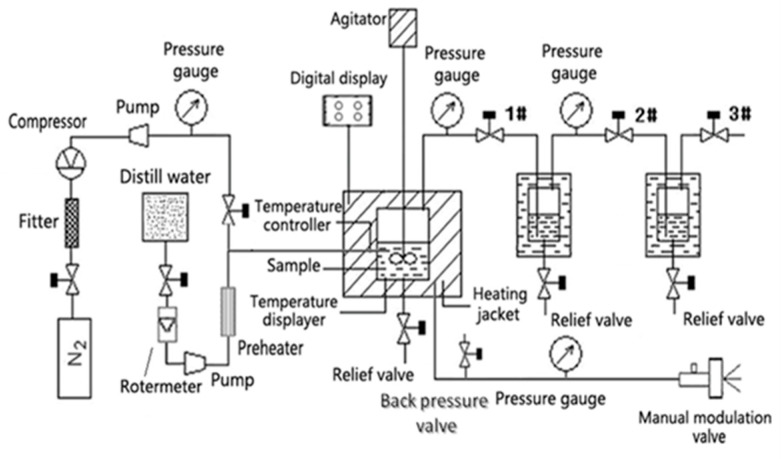
The schematic diagram of subcritical reaction system.

SWE was carried out in an apparatus (Model CWYF-2; Sihai Instrument Inc. Nantong, China), and a schematic diagram is shown in Figure 7. According to Fan’s report [38], the extraction procedure was as follows: (1) A sample was loaded into a stainless steel vessel (inner volume: 300 mL) packed in an oven with a heating jacket connected to a temperature and pressure sensor, and the parameters were shown on the digital display meters. (2) After being tightly closed with a stainless steel cap, a constant flow rate of deionized water was purged via a syringe pump into the extraction vessel, while using nitrogen to remove dissolved oxygen before the extraction. A magnetic stirrer was activated to improve the mass transfer. Pressure was regulated by a back pressure regulating valve. Static extraction was performed (i.e., 20 min). (3) The extraction process was finished before depressurization took place.

SWE experiments were carried out under a constant pressure of 7.0 MPa, and each experiment lasted for 20 min under different temperatures (80–340 °C). Five grams of licorice powder was loaded for extraction with a solid-to-water ratio of 1:30 and a stirring speed of 150 rpm. The crude extract was collected and cooled down to ambient temperature, and then classified into three parts: aqueous solution, ethanolic solution, and residue. The phase isolation procedure was performed by the method reported by Fan et al. [10]. The cooling crude extract was centrifuged at 1500× *g* for 10 min, the supernatant (aqueous solution) was collected, then 30 mL of ethanol (60% *v*/*v*) was used to redissolve the sediment, which was insoluble in water, at room temperature. It was shaken and then centrifuged at 1500× *g* for 10 min. The supernatant (ethanolic solution) was separated and collected; the final sediment, which could have been wax, hemicellulose, and other undesired material, was discarded. The contents of bioactive compounds including total phenolics, total flavonoids, glycyrrhizic acid, glycyrrhetinic acid and Glycyrrhetinic acid 3-*O*-mono-β-D-glucuronide in the extracts were determined from the two parts (aqueous and ethanolic phase).

### 3.3. Determination of the Bioactive Compounds

#### 3.3.1. The Total Phenolic and Total Flavonoid Content

The total phenolic content in the extract (including the aqueous and ethanolic phases) was estimated according to the method described by Singleton [39]. Briefly, 0.5 mL of diluted extract was mixed with 2.5 mL of Folin–Ciocalteu reagent (0.2 mol/L). After 10 min, 2 mL of a sodium carbonate solution (7.5%, *w*/*v*) was added. The mixture was vortexed and stood for 2 h at room temperature. The absorbance was measured at 760 nm spectrophotometrically. Results were calculated on the basis of the calibration curve of gallic acid and expressed as gallic acid equivalents (g GAE/100 g dry basis raw materials).

Total flavonoid content in the extract (including the aqueous and ethanolic phases) was determined by the aluminum chloride colorimetric method [40]. Briefly, 0.5 mL of a sample (appropriately diluted) was placed in a 10 mL graduated test tube, and 3.5 mL of distilled water and 0.3 mL of sodium nitrite (7.5 g/100 mL) were added and mixed. Three milliliters of an aluminum chloride solution (1 g/100 mL) was added 5 min later, and 2 mL of a sodium hydroxide solution (4 g/100 mL) was then added. The total volume was adjusted to 10 mL with distilled water and vibrated for 20 s. After 15 min, the absorbance was measured at 510 nm spectrophotometrically. Total flavonoid content was calculated from a calibration curve of rutin and expressed as rutin equivalent (g RE/100 g dry basis raw materials).

#### 3.3.2. Determination of Glycyrrhizic Acid and Its Hydrolytic Products

The content of glycyrrhizic acid and its hydrolytic products was measured using HPLC-DAD (Agilent, American) with a Zorbax Eclipse Plus C18 column (250 × 4.6 mm, i.d., 5 μm) [21]. The mobile phase consisted of Solvent A (100% methanol) and Solvent B (1.0% acetic acid in an ammonium acetate solution) at a ratio of 80:20. The flow rate was 0.8 mL/min, the column temperature was kept at 35 °C, and the injector volume was 20 μL. The DAD wavelength was set at 250 nm.

### 3.4. Evaluation of Radical Scavenging Capacity

The scavenging capacity of the extract towards the DPPH· assay was monitored according to the methods of Ramadan (2003) [41]. The DPPH· solution was freshly prepared in methanol at a concentration of 1.75 × 10−hemol/L. About 2.0 mL of the DPPH· solution was added to 2.0 mL of the appropriately diluted extracts and vibrated for 20 s at room temperature. The absorbance of the mixture was spectrophotometrically recorded at 517 nm after reacting for 1 h in the dark. A control, in which the sample was replaced by methanol, was measured in the same way. The antioxidant activity of the extracts was calculated from the dose–response curves of Trolox, which were determined by the above-mentioned methods. All of the results were expressed as the Trolox equivalent antioxidant capacity (mmol TEAC/g dry basis raw materials).

The scavenging capacity of the extract towards the ABTS·+ assay were monitored according to the methods of Pellegrini (2001) [42]. Briefly, 10 mg of ABTS was diluted in 2.6 mL of a potassium persulfate solution (2.45 mM) and kept in the dark at 4 °C for 12 h, and the mixture was then diluted to an absorbance of 0.70 ± 0.02 to be used as an ABTS·+ work solution. A 1 mL sample (appropriately diluted) and a 3 mL ABTS·+ work solution were mixed and reacted for 60 min at room temperature. The absorbance was spectrophotometrically measured at 734 nm. A control, in which the sample was replaced by methanol, was measured in the same way. The antioxidant activity of the extracts was calculated from the dose–response curves of Trolox, which were determined by the above-mentioned methods. All of the results were expressed as the Trolox equivalent antioxidant capacity (mmol TEAC/g dry basis raw materials).

### 3.5. HPLC-ABTS^+^ and Mass Spectrometric Analysis of Bioactive Compounds in the Extracts

The bioactive compounds in the **licorice** extract were analyzed with HPLC-DAD. Chromatographic analysis was run using a reverse-phase Zorbax XDB C-18 (150 × 4.6 mm, i.d., 5 μm), and the extract was separated using two solutions, A (methanol) and B (1.0% acetic acid in water, v/v). Gradient elution was performed as follows: 0 min: 10% A; 8 min: 10% A; 15 min: 20% A; 28 min: 25% A; 35 min: 30% A; 40 min: 36% A; 45 min: 37% A; 50 min: 30% A; 58 min: 38% A; 70 min: 58% A; 100 min: 82% A; 105 min: 100% A; 107 min: 100% A. The flow rate was 0.9 mL/min, the temperature was kept at 35 °C, and the injector volume was 10 μL. The DAD wavelengths were set at 250, 280, and 360 nm, respectively.

While the bioactive compounds in the extract were separated in the column, an ABTS·^+^ work solution was delivered via a pulse pump (Waters Corporation, Milford, MA, USA) with a flow rate of 0.7 mL/min. The ABTS·+ work solution was mixed with the fractions from the column and reacted in the peak coil. The mixture was then transported into the DAD detector to monitor the absorbance at 734 nm, and signals of the positive and negative peaks were recorded [38].

In order to identify the active compounds in the extract, HPLC-MS/MS fitted with an electrospray ionization (ESI) source was used. The HPLC separation program of different fractions from the extract was the same as described above; total ion current (TIC) chromatograms were acquired in both positive and negative modes. The electrospray capillary voltage was set to 3500 V. Nitrogen was used as the nebulizing gas at a pressure of 30 psi with a flow rate of 9.0 L/min and drying gas temperature was 325 °C. The scan range of the mass spectrometry was *m*/*z* 50–1500.

### 3.6. Determination of Advanced Glycated End Products

In order to check the formation of the advanced glycation end products in the SWE extracts, the fluorescence of the extracts was measured using a fluorospectrophotometer (F-7000; Hitachi, Japan). Advanced glycation end products were analyzed according to the method reported by Plaza et al. (2010) with a minor modification [34]. The excitation wavelength (*λ_Ex_*) was 360/40 nm, and the emission wavelengths were 460/40 and 528/20 nm, respectively.

### 3.7. Analysis of Non-Specific Indicators of Maillard Reaction

#### 3.7.1. Determination of Total Reducing Sugar Content

The total reducing sugar content was analyzed according to 3,5-dinitrosalicylicacid (DNS) colorimetric method [43]. Briefly, 0.5 mL of diluted extract was mixed with 0.5 mL of DNS reagent and placed in boiling water for 5 min; after the mixture was cooled to room temperature in a water bath, distilled water was added to a given volume of 5.0 mL. The absorbance was measured at 540 nm. D-Glucose was used as a standard, and the result was expressed in g/100 g dry basis raw materials.

#### 3.7.2. Determination of Total Free Amino Acid Content

The total free amino acid content was analyzed according to the OPA assay reported by Chen et al. (2009) [44] and the Chinese Standard GB/T8314-87. Briefly, 1.0 mL of diluted extract was mixed with 0.5 mL of phosphate buffer (pH8.0) and 0.5 mL of 0.2% ninhydrin solution (containing 0.8 mg/mL SnCl2). The mixture was then heated in boiling water for 15 min, cooled to room temperature, and adjusted to 25 mL with distilled water. Absorbance at 568 nm was measured with a spectrophotometer. The result was expressed in l-glutamic acid equivalent, g/100 g dry basis raw materials.

#### 3.7.3. Measurement of Color

It is widely accepted that the development of a brown color can be effectively used to monitor the occurrence of non-enzymatic browning reactions, including the Maillard reaction [32]. In fact, this color is the easiest way to measure the existence of MRPs, as it is a visual estimation. The color of the extract was determined using a Chroma Meter DC-P3 optical sensor (Beijing Xingguang Color-Measurement Instrument Co., Ltd., Beijing. China). One of the most common methods employed for color measurement related to browning intensity was proposed by Delgado-Andrade (2010) [33]. The yellowing index (YI) is calculated as follows:

YI = 142.86b*/L* (1)

#### 3.7.4. Absorbance Measurement

Melanoidins were estimated by means of the browning intensity of the extracted samples. Browning intensity was measured using a microplate spectrophotometer. According to a previous report [23], absorbance at 280, 360, and 420 nm was measured to monitor products in the early, more advanced, and final stages of the Maillard reaction (Shimadzu UV-1800, Kyoto, Japan).

### 3.8. Analysis of Specific Indicators of Maillard Reaction

The compound 5-Hydroxymethyl furfural is an important intermediate compound produced in the Amadori rearrangement of a Maillard reaction. The Maillard reaction products, 5-hydroxymethyl furfural (5-HMF) and furfural, were analyzed using HPLC-DAD (Agilent, American) with a Zorbax XDB C-18 column (250 × 4.6 mm, i.d., 5 μm). The injected volume was 20 μL, and the temperature and wavelength were set to 35 °C and 280 nm. The 5-HMF content was eluted with a mobile phase of methanol/water (10:90, *v*/*v*) at a constant flow of 0.6 mL/min, and furfural was eluted with a mobile phase of acetonitrile/water (2:98, *v*/*v*) at a constant flow of 1 mL/min [38].

### 3.9. Kinetic Analysis of Subcritical Water Extraction for Glycyrrhizic Acid

The glycyrrhizic acid extraction process could be viewed as an irreversible, consecutive, unimolecular-type first-order mechanism, in which glycyrrhizic acid is firstly extracted from licorice by diffusion following a first-order-like mechanism [45], and glycyrrhizic acid in the extract is then subjected to a hydrolytic reaction following a first-order mechanism due to the extension of extraction time under a high temperature and pressure [46]. Accordingly, the glycyrrhizic acid extraction process can be simplified into the following reaction scheme:
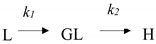


where, L and GL represent the content of glycyrrhizic acid in licorice and extract, respectively; H represents the content of the hydrolytic product in the extract; and *k_1_* and *k_2_* represent the rate constants for the extraction and hydrolytic steps, respectively.

The following simplified reaction models with a minor modification were used for estimating the rate constant for glycyrrhizic acid extraction and hydrolysis [41]:

(2) $$\frac{dm_{L}}{dt}=-k_{1}m_{L}$$

(3) $$\frac{dm_{GL}}{dt}=k_{1}m_{L}-k_{2}m_{GL}$$

(4) $$\frac{dm_{H}}{dt}=k_{2}m_{GL}$$

where *m_L_* (g) and *m_GL_* (g) are the glycyrrhizic acid content in licorice and extract at time *t*, and *m_H_* (g) is the content of hydrolytic product in the extract at time *t*; *t* (s) is the extraction time; *k_1_* and *k_2_* (s^−1^) is the rate constant for glycyrrhizic acid extraction and hydrolysis.

After a rearrangement, Equation (2) was shown as Equation (5):

(5) $$\ln(\frac{m_{L}}{m_{L0}})=k_{1}t\;\mathrm{or}\;m_{L}=m_{L0}e^{-k_{1}t}$$

*m_L_*_0_ (g) is the initial content of glycyrrhizic acid in licorice at time *t* = 0, where *k_1_* (s^−1^) is the rate constant for glycyrrhizic acid extraction.

To investigate the variation in glycyrrhizic acid content, substitute Equation (2) into Equation (3):

(6) $$\frac{dm_{GL}}{dt}=k_{1}m_{L0}e^{-k_{1}t}-k_{2}m_{GL}$$

By integration of Equation (6), the final variation in glycyrrhizic acid content was expressed as:

(7) $$m_{GL}=\frac{m_{L0}k_{1}}{k_{2}-k_{1}}(e^{-k_{1}t}-e^{-k_{2}t})$$

Equation (7) was also expressed as the yield (g/g):

(8) $$yield_{GL}=\frac{k_{1}}{k_{2}-k_{1}}(e^{-k_{1}t}-e^{-k_{2}t})$$

The extraction and hydrolytic rates were accurately described by first-order kinetics, the relationship between rate constants and the temperature was estimated by the Arrhenius law with *Ea* and *A* (Equation (9)), *A* is the pre-exponential factor and *Ea* is the activation energy:

(9) $$k=A\exp(-\frac{Ea}{RT})$$

and, after a rearrangement:

(10) $$\ln k=-\frac{Ea}{RT}+\ln A$$

Accordingly, these kinetic parameters were estimated by plotting ln *k* versus *T*.

### 3.10. Statistical Analysis

All of the experiments and measurements were carried out in triplicate. Analysis of variance (ANOVA) was carried out by SPSS 16.0 software, and significant differences in means (*p* < 0.05) were determined using Duncan’s multiple-range test. The fitting equation was estimated with Origin 8.0.

## 4. Conclusions

The maximum contents of total flavonoids, phenolics and glycyrrhizic acid was extracted from licorice with subcritical water at 140 °C for 20 min. With the rise in temperature, the Maillard reaction occurred; thus, the Maillard reaction contributed to the antioxidant activity of the extract. In addition, the hydrolysis of glycyrrhizic acid occurred during the extraction process. Glycyrrhizic acid was first extracted by diffusion following a first-order mechanism and was then hydrolyzed following a first-order mechanism during SWE. It was discovered in this study that subcritical water can be used as a medium for hydrolyzing glycyrrhizic acid into glycyrrhetinic acid 3-O-mono-β-D-glucuronide and glycyrrhetinic acid. Therefore, SWE is not only an environmentally friendly processing technology but also a highly efficient method for producing bioactive compounds.

## Figures and Tables

**Table 1:** The fluorescence, color, and absorbance of licorice extracts obtained at different temperatures.

Temperature (°C)	Fluorescence		Color				Absorbance		
	λ_Ex_ 360 nm, λ_Em_ 460 nm	λ_Ex_ 360 nm, λ_Em_ 528 nm	L*	a*	b*	Yellowing Index	*A*_280nm_	*A* _360nm_	*A* _420nm_
80	22.021 ± 1.410 ^a^	2.512 ± 0.722 ^a^	21.992 ± 0.290 ^d^	1.981 ± 0.283 ^a^	10.345 ± 1.202 ^a^	67.301 ± 8.721 ^a^	0.126 ± 0.010 ^a^	0.032 ± 0.009 ^a^	0.003 ± 0.001 ^a^
100	22.200 ± 0.720 ^a^	2.521 ± 0.720 ^a^	20.991 ± 1.401 ^c^	3.502 ± 1.412 ^ab^	11.333 ± 0.472 ^ab^	77.182 ± 1.972 ^a^	0.132 ± 0.009 ^a^	0.039 ± 0.005 ^a^	0.004 ± 0.001 ^a^
120	20.801 ± 0.710 ^a^	2.503 ± 0.711 ^a^	17.923 ± 1.942 ^c^	3.740 ± 0.713 ^abc^	11.511 ± 0.012 ^abc^	92.292 ± 9.871 ^b^	0.466 ± 0.001 ^c^	0.154 ± 0.009 ^e^	0.019 ± 0.002 ^b^
140	51.201 ± 1.411 ^b^	12.512 ± 0.722 ^b^	13.362 ± 0.511 ^b^	5.090 ± 1.410 ^bcd^	11.201 ± 0.711 ^ab^	119.702 ± 3.002 ^c^	0.901 ± 0.008 ^f^	0.348 ± 0.003 ^h^	0.068 ± 0.002 ^c^
160	79.402 ± 1.401 ^c^	26.512 ± 0.714 ^c^	13.411 ± 0.582 ^b^	5.751 ± 0.851 ^cd^	11.923 ± 0.410 ^abcd^	127.302 ± 9.723 ^c^	1.309 ± 0.002 ^h^	0.463 ± 0.003 ^j^	0.145 ± 0.002 ^d^
180	115.300 ± 1.412 ^d^	41.221 ± 1.421 ^d^	11.770 ± 1.073 ^ab^	6.071 ± 1.362 ^d^	11.682 ± 1.122 ^abcd^	141.801 ± 0.711 ^d^	1.766 ± 0.005 ^m^	0.569 ± 0.001 ^k^	0.178 ± 0.001 ^e^
200	244.511 ± 2.111 ^e^	90.011 ± 1.412 ^g^	11.593 ± 0.423 ^ab^	6.522 ± 0.422 ^d^	12.061 ± 0.272 ^bcd^	148.811 ± 8.762 ^de^	1.704 ± 0.013 ^l^	0.571 ± 0.004 ^k^	0.201 ± 0.001 ^f^
220	346.012 ± 1.422 ^h^	120.012 ± 1.423 ^h^	10.692 ± 1.402 ^a^	6.331 ± 1.703 ^d^	11.881 ± 1.072 ^abcd^	159.192 ± 6.491 ^ef^	1.611 ± 0.008 ^k^	0.576 ± 0.009 ^k^	0.221 ± 0.009 ^g^
240	532.512 ± 3.513 ^j^	164.311 ± 2.82 ^j^	10.942 ± 0.733 ^a^	6.332 ± 0.621 ^d^	11.662 ± 0.801 ^abcd^	152.263 ± 0.110 ^def^	1.324 ± 0.002 ^i^	0.432 ± 0.011 ^i^	0.224 ± 0.011 ^g^
260	581.812 ± 1.423 ^k^	180.211 ± 1.401 ^k^	10.941 ± 0.361 ^a^	6.542 ± 2.020 ^d^	12.472 ± 0.662 ^bcd^	162.782 ± 3.211 ^f^	1.343 ± 0.001 ^j^	0.2875 ± 0.007 ^g^	0.230 ± 0.009 ^g^
280	684.502 ± 3.512 ^l^	202.012 ± 2.812 ^l^	12.050 ± 1.202 ^ab^	7.071 ± 1.232 ^d^	13.231 ± 0.181 ^d^	157.522 ± 13.532 ^ef^	1.101 ± 0.001 ^g^	0.233 ± 0.003 ^f^	0.222 ± 0.009 ^g^
300	484.311 ± 5.621 ^i^	134.212 ± 2.800 ^i^	11.223 ± 0.132 ^a^	7.282 ± 1.531 ^d^	12.682 ± 0.681 ^bcd^	161.701 ± 6.822 ^ef^	0.543 ± 0.003 ^e^	0.137 ± 0.004 ^d^	0.169 ± 0.015 ^e^
320	281.221 ± 1.415 ^g^	75.524 ± 0.712 ^e^	11.041 ± 0.131 ^a^	7.011 ± 1.423 ^d^	12.412 ± 1.833 ^bcd^	160.312 ± 4.712 ^ef^	0.399 ± 0.001 ^b^	0.099 ± 0.003 ^b^	0.138 ± 0.010 ^d^
340	264.502 ± 0.732 ^f^	70.511 ± 0.712 ^f^	11.692 ± 0.742 ^ab^	7.132 ± 0.183 ^d^	13.112 ± 0.331 ^cd^	160.412 ± 6.110 ^ef^	0.483 ± 0.005 ^d^	0.112 ± 0.009 ^c^	0.062 ± 0.002 ^c^

The results are expressed as the mean ± standard deviation (*n* = 3); the different letters indicate that the difference was significant (*p* < 0.05) and the same letters indicate insignificant differences.

## Data Availability

The data are not publicly available due to graduation thesis based on this relevant research results is still in the confidentiality period.

## Supplementary Materials

The following supporting information can be downloaded at: https://www.mdpi.com/article/10.3390/molecules27206851/s1, Figure S1: UPLC-MS profiles of bioactive compounds in the liquorice extract and their corresponding standards, the scanning spectra of bioactive compounds. Figure S2: The structures of flavonoids in liquorice; Table S1: Correlation analysis between TP, TF contents and antioxidant activity of liquorice extracts in different range of temperature; Table S2: The effect of extraction temperature on the content of total flavonoids, total phenolics and total antioxidant activity.
