# Supplementary material for: Maillard and Hydrolytic Reactions in Subcritical Water Extraction of Bioactive Compounds from Licorice
Source: Molecules. 2022 Oct 13;27(20):6851. doi: 10.3390/molecules27206851 (PMC9607042; doi:10.3390/molecules27206851)
Supplement: Supplementary file 1 [file molecules-27-06851-s001.zip › molecules-1864994-supplementary.pdf]

The Maillard and hydrolytic reactions in subcritical water extraction of bioactive compounds from liquorice

Running title: The reactions in subcritical water extraction of bioactive compounds from liquorice

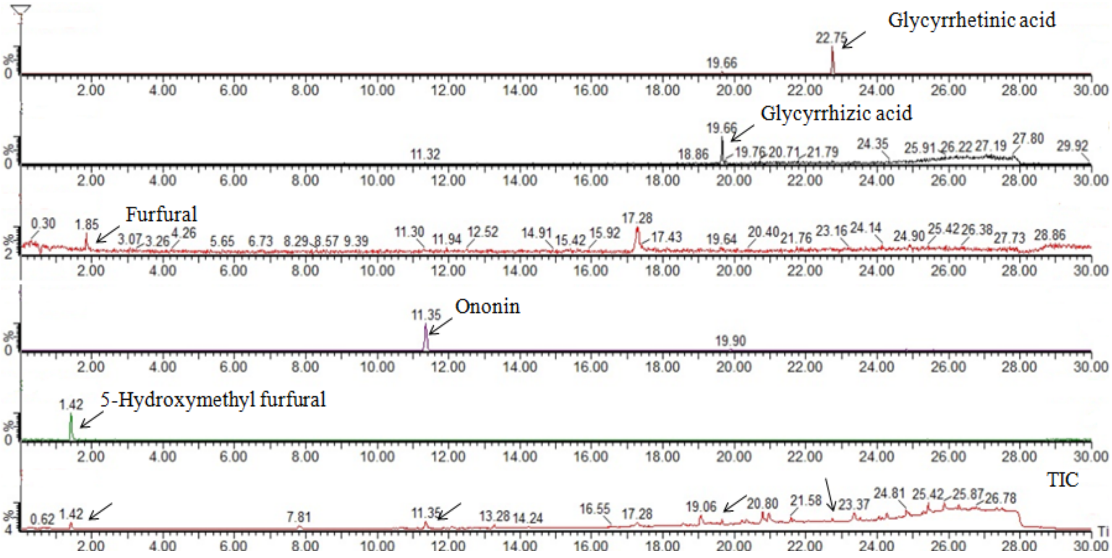

(a)

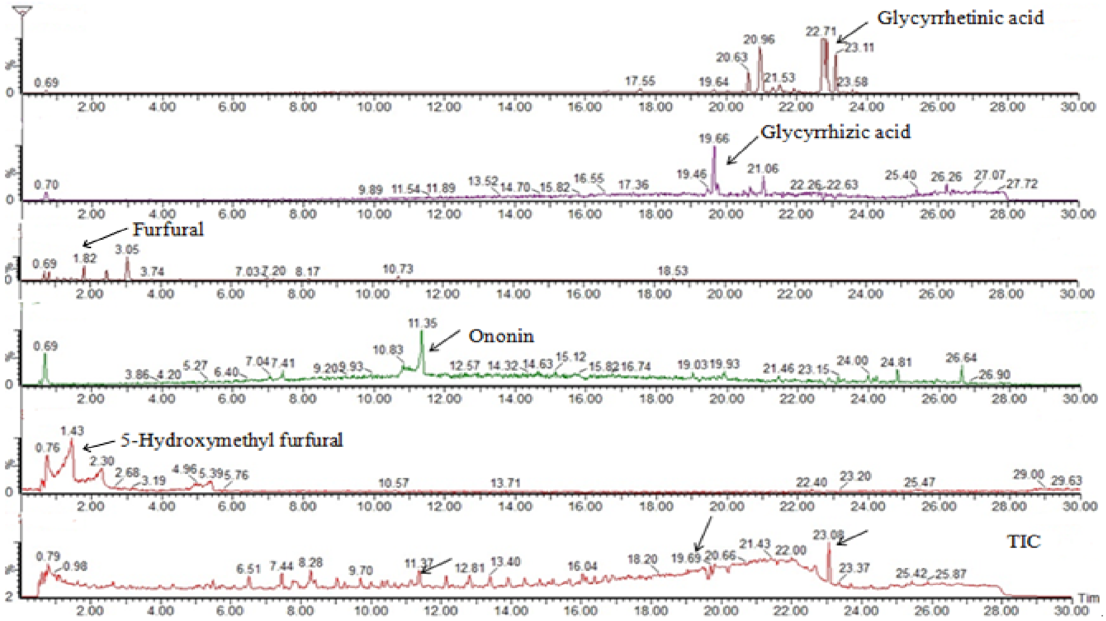

(b)

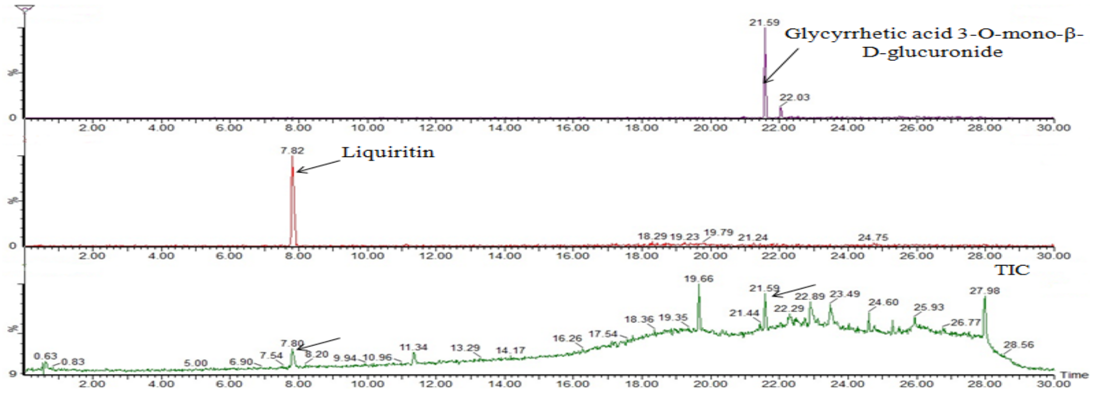

(c)

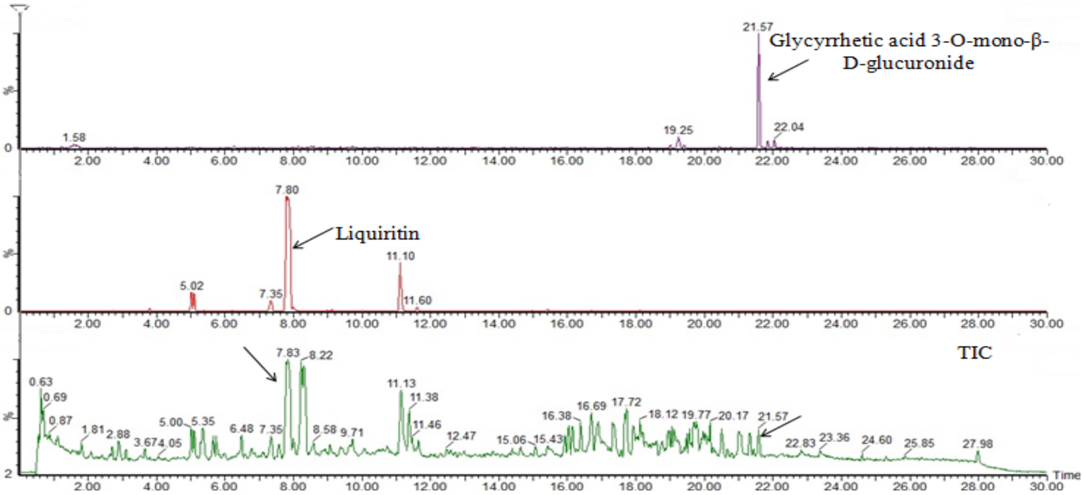

(d)

Figure S1. UPLC-MS profiles of bioactive compounds in the liquorice extract and their corresponding standards, the scanning spectra of bioactive compounds (a) and their corresponding standards (b) under the nagative ion mode; the scanning spectra of bioactive compounds (c) and their corresponding standards (d) under positive ion mode.

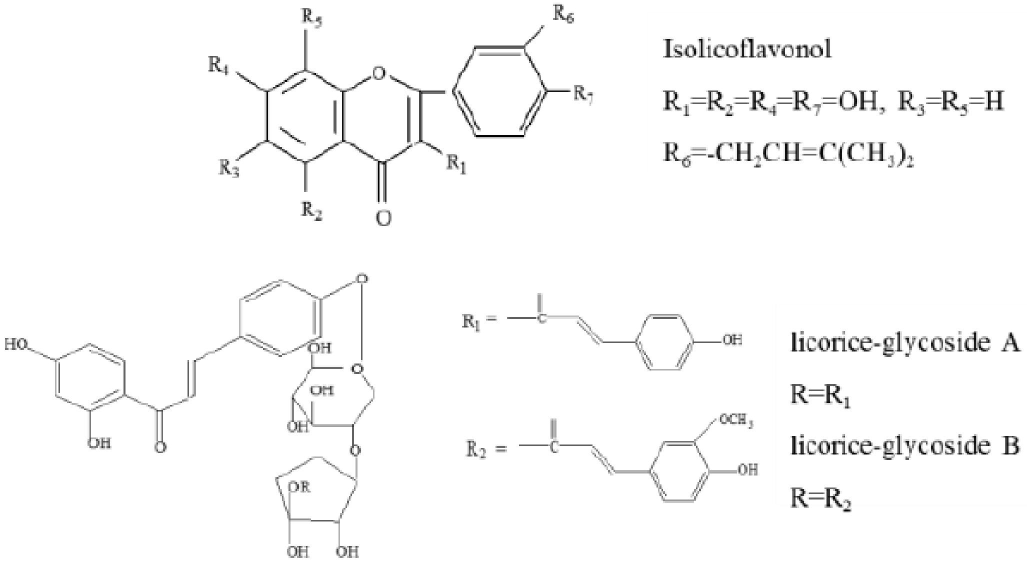

Figure S2. The structures of flavonoids in liquorice

Table S1. Correlation analysis between TP, TF contents and antioxidant activity of liquorice extracts in different range of temperature

Temperature range (°C)	ABTS-TP	ABTS-TF	DPPH-TP	DPPH-TF
80-240 °C	Y=0.124x-0.017	Y=0.262x-0.053	Y=0.15x-0.046	Y=0.07x-0.024
	R^2=0.941	R^2=0.943	R^2=0.935	R^2=0.920
240-280 °C	Y=-0.002x+0.506	Y=-0.405x+1.827	Y=-0.23x+0.738	Y=-0.285x+1.22
	R^2=0.005	R^2=0.852	R^2=0.083	R^2=0.889

Table S2. The effect of extraction temperature on the content of total flavonoids, total phenolics and total antioxidant activity.

	Contents (g/100g dry basis)		Trolox equivalent antioxidant capacities (mmol/g dry basis)	
Temperature(℃)	Total phenolics	Total flavonoids	DPPH	ABTS
80	0.253±0.002[a]	0.235±0.007[a]	0.013±0.001[a]	0.029±0.001[a]
100	0.310±0.004[b]	0.291±0.0001[b]	0.015±0.001[a]	0.036±0.001[a]
120	1.190±0.002[c]	0.721±0.003[c]	0.028±0.001[b]	0.050±0.002[b]
140	1.890±0.003[g]	1.025±0.003[d]	0.095±0.002[c]	0.238±0.010[c]
160	3.004±0.006[h]	1.465±0.097[g]	0.153±0.002[e]	0.388±0.001[e]
180	4.060±0.003[l]	1.977±0.048[hi]	0.211±0.005[f]	0.396±0.001[e]
200	4.230±0.001[n]	1.954±0.005[hi]	0.300±0.001[hi]	0.516±0.018[h]
220	4.081±0.010[m]	2.162±0.011[k]	0.295±0.004[h]	0.513±0.004[h]
240	3.391±0.001[k]	1.985±0.004[i]	0.255±0.002[g]	0.459±0.003[f]
260	3.240±0.002[j]	1.923±0.001[h]	0.294±0.005[h]	0.498±0.002[g]
280	3.223±0.020[i]	2.038±0.044[j]	0.309±0.010[i]	0.544±0.002[i]
300	1.562±0.004[f]	1.341±0.001[f]	0.135±0.002[d]	0.273±0.003[d]
320	1.462±0.005[e]	1.145±0.001[e]	0.126±0.022[d]	0.269±0.015[d]
340	1.442±0.003[d]	1.150±0.002[e]	0.136±0.002[d]	0.270±0.008[d]

The results were expressed as mean ± standard deviation (n=3), the different letters indicated that the difference was significant ($P < 0.05$) and the same letter was expressed as insignificant difference
